# Differences in Lower‐Limb Kinematic–Electromyographic Coupling Patterns During Volleyball Spike Take‐Off Between Outside Hitters and Middle Blockers

**DOI:** 10.1155/abb/2870235

**Published:** 2026-10-01

**Authors:** Chen Chen, Jian Yu, Yang Wang, Cheng Chen, Youn-poong Oh, Shuangrui Liu, Zhenhao Lin, Yuwen ShangGuan

**Affiliations:** ^1^ Department of Physical Education and Aesthetic Education Shandong Polytechnic Jinan 250104 Shandong China; ^2^ Physical Education Department Kunsan National University Gunsan 541150 South Korea kunsan.ac.kr; ^3^ Department of Physical Education Shandong University of Traditional Chinese Medicine Jinan China sdutcm.edu.cn; ^4^ Graduate School Harbin Sport University Harbin China hrbipe.edu.cn; ^5^ Institute of Student Physical Health Promotion Jingchu University of Technology Jingmen 448000 Hubei Province China jcut.edu.cn; ^6^ Department of Physical Education Graduate School Kyungpook National University Daegu 41566 South Korea knu.ac.kr; ^7^ School of Rehabilitation Medicine Jining Medical University Jining Shandong China jnmc.edu.cn

**Keywords:** approach jump, kinematic–electromyographic coupling patterns, lower-limb kinematics, middle blocker, outside hitter, surface electromyography (EMG)

## Abstract

**Background:**

This study compared lower‐limb kinematics and electromyographic characteristics between outside hitters (OH) and middle blockers (MB) during volleyball spike take‐off, aiming to delineate position‐specific joint‐angle profiles and relative muscle activation contributions.

**Methods:**

Twelve national first‐class male volleyball athletes were recruited (OH, *n* = 6; MB, *n* = 6), and three‐dimensional kinematics (240 Hz) and surface electromyography (EMG; 2000 Hz) were synchronously collected. The analysis window was defined from right‐foot contact in the final approach step to bilateral toe‐off (T1–T4), with the braking phase set as T1–T3 and the propulsion phase as T3–T4; T3 was treated as the transition boundary between the two phases rather than as an independent phase. All participants were right‐handed hitters; therefore, the right side was defined as the dominant hitting side and the left side as the non‐dominant/non‐hitting side. Sagittal‐plane hip, knee, and ankle joint angles and phase‐specific relative iEMG contribution percentages of seven muscles were calculated and compared between groups.

**Results:**

OH exhibited a smaller right/dominant hitting‐side hip angle than MB at T1, indicating a more flexed sagittal‐plane hip posture (*p* < 0.01), and a smaller left/non‐dominant hitting‐side hip angle during T2–T3 (*p* < 0.05). The right/dominant hitting‐side knee angle was consistently smaller in OH than in MB throughout T1–T3, indicating greater knee flexion (*p* < 0.01). For the ankle joint, OH showed a greater left/non‐dominant hitting‐side sagittal‐plane ankle included angle during T1–T3 (*p* < 0.01), whereas MB showed a greater right/dominant hitting‐side sagittal‐plane ankle included angle at T2 and T3 (*p* < 0.01). During braking, OH showed a right/dominant hitting‐side iEMG contribution of 68.90% and a left/non‐dominant hitting‐side contribution of 31.09%, whereas MB showed 65.20% and 34.79%, respectively. During propulsion, OH primarily relied on the left/non‐dominant hitting side (59.40% vs. 40.60%), whereas MB demonstrated a more balanced bilateral activation pattern (49.63% vs. 50.37%).

**Conclusion:**

OH and MB exhibited distinct lower‐limb kinematic–EMG coupling patterns during spike take‐off. OH showed greater sagittal‐plane hip and knee flexion in the early take‐off phase and a force‐production shift from right/dominant hitting‐side braking to left/non‐dominant hitting‐side propulsion, whereas MB demonstrated relatively larger joint angles and a more balanced bilateral activation pattern. These findings support position‐specific take‐off technique training and neuromuscular control strategies.

## 1. Introduction

The volleyball spike is one of the key determinants of offensive efficiency [1, 2], and its successful execution relies heavily on lower‐limb explosive power and neuromuscular coordination [3, 4]. From a biomechanical perspective, the spike take‐off can be divided into two phases [5]: the approach phase, which primarily generates horizontal velocity, and the take‐off phase, in which multi‐joint coordination of the lower limbs converts horizontal velocity into vertical velocity [6, 7]. As the core transitional link for horizontal‐to‐vertical momentum conversion, the final approach step directly influences jump height and the conditions for ball contact [8–10]. Previous studies indicate that jump height is closely associated with approach speed, take‐off angle, and key kinematic characteristics of the knee and ankle joints [11, 12].

From the perspective of multi‐joint coordination of the lower limbs, the spike take‐off is a typical multi‐joint‐linked movement: at the end of the approach, braking and reacceleration must be completed within a short time, and the shift in the direction of propulsion is achieved through sequential flexion–extension coordination of the hip–knee–ankle during ground contact, braking, and propulsion [13]. Because the control roles of each joint change dynamically over time, characterizing multi‐joint coordination using a time‐series and phase‐based framework is more conducive to reflecting the phase‐specific control demands of volleyball‐specific movements [14]. In comparisons of different landing strategies after spiking, studies integrating time‐series kinematics and kinetics and applying statistical parametric mapping (SPM) have shown that key differences are concentrated in the high‐risk time window during early landing, highlighting the importance of a “temporal–phase” perspective for understanding the control mechanisms of volleyball‐specific movements [15]. Compared with the landing phase, evidence regarding multi‐joint temporal coordination during the critical transition segment of spike take‐off (particularly from final‐step foot contact to bilateral toe‐off) and its synchronous relationship with the distribution of muscle activation remains relatively limited.

With the improvement of the competitive level, position specialization in volleyball has become increasingly pronounced [16]. As key offensive positions, outside hitters (OHs) and middle blockers (MBs) differ in technical requirements and tactical responsibilities [17, 18]. OH typically undertake power attacks and serve reception, and they need to organize more complex approach–take‐off sequences during offense–defense transitions [19, 20]; in contrast, MB focus primarily on rapid blocking and quick attacks, with a faster movement rhythm and a higher jump frequency [21]. Previous studies suggest that athletes in different positions may exhibit distinct hip, knee, and ankle coordination characteristics and muscle activation patterns during take‐off [22]. For example, OH may require greater hip flexion, whereas MB may show more pronounced knee extension angular velocity and ankle dorsiflexion characteristics [23, 24]. In addition, bilateral asymmetry in neuromuscular distribution may still exist during two‐foot take‐off [25–27], and different take‐off techniques may correspond to different activation strategies. Although existing evidence indicates that positional and technical factors affect take‐off control, systematic comparisons in elite athletes of the synchronous coupling between “multi‐joint temporal coordination” and “muscle activation distribution” during the critical transition segment of spike take‐off remain limited.

Therefore, this study addresses the following question. Under position‐typical spiking conditions, do OH and MB exhibit position‐specific kinematic–electromyographic coupling differences in (i) the temporal changes in hip, knee, and ankle joint angles and (ii) the activation distribution of the primary muscles during the take‐off transition segment (T1–T4)? To address this question, three‐dimensional kinematic and surface electromyography (EMG) data were synchronously collected. Temporal features of hip, knee, and ankle joint angles and the relative iEMG contribution percentages of seven primary muscles were calculated for the braking phase (T1–T3) and the propulsion phase (T3–T4), and coupling comparisons were conducted within a unified phase‐based framework to characterize position‐related lower‐limb coordination patterns. This study aims to provide position‐specific training references based on synchronized kinematic–electromyographic evidence. The scope of this study is limited to kinematic and electromyographic outcomes, and no direct inferences are made regarding kinetic variables such as joint moments or power.

## 2. Methods

### 2.1. Participants

This study recruited 12 national first‐class male volleyball athletes, including six OH and six MB. All participants were right‐handed hitters and performed the spike using the right arm. Therefore, in this study, the right side was operationally defined as the dominant‐hitting side, whereas the left side was defined as the non‐dominant/non‐hitting side. Because lower‐limb dominance was not independently assessed, the terms “dominant” and “non‐dominant” refer specifically to the hitting‐arm side rather than to the independently measured lower‐limb dominance. Accordingly, lower limb dominance was not entered as an independent grouping factor or covariate in the analysis. Participants’ age, height, body mass, body mass index (BMI), and years of training were recorded prior to testing to describe baseline characteristics between groups (Table 1). Previous injury history was confirmed via questionnaires and interviews to exclude diseases or injuries that could affect lower‐limb motor function. Participants avoided strenuous exercise and alcohol for 24 h before testing and consumed a meal 2 h before the test session. A significant between‐group difference in height was observed. To reduce the potential confounding influence of anthropometric differences (particularly height) on between‐group comparisons, the covariate‐control and sensitivity analysis strategies are described in Section 2.5 (Statistical analysis) and Table S1.

**Table 1:** Participant characteristics (mean ± standard deviation).

Variable	Outside hitter (OH, *n* = 6) mean ± SD	Middle blocker (MB, *n* = 6) mean ± SD	*p*‐Value
Height (cm)	186.75 ± 4.99	195.30 ± 3.10	0.007
Body mass (kg)	78.75 ± 7.89	85.60 ± 5.40	0.114
Age (year)	23.25 ± 1.26	22.50 ± 1.61	0.458
Years of training (year)	7.83 ± 2.14	8.17 ± 1.47	0.760
Body mass index (BMI/kg/m^2^)	22.52 ± 1.06	22.42 ± 0.71	0.852

*Note:* The *p*‐values are results from independent‐samples *t*‐tests.

Inclusion criteria: ≥5 years of systematic volleyball training; weekly training duration ≥16 h; no acute or chronic injuries affecting performance within the past 12 months; no neurological diseases affecting lower‐limb motor function; BMI between 18.5 and 25.0 kg/m^2^.

Exclusion criteria: severe sports injuries within the past 6 months requiring >2 weeks of rest; pain or other discomfort during testing that interfered with performance; chronic diseases or long‐term medication use that could influence performance.

Written informed consent was obtained from all participants prior to the experiment. This study followed the ethical principles of the Declaration of Helsinki and was approved by the Research Ethics Committee of Yanshan University (March 2, 2022).

### 2.2. Experimental Design

This experiment was conducted under a match‐like scenario with position‐typical spiking tasks [2, 4]. The same setter stood at a fixed location between Zone 2 and Zone 3 to deliver sets for both positions. OH performed power spikes from Zone 4, whereas MB performed quick attacks from Zone 3. Because the two positions differ in approach rhythm and step patterns in competition (OH commonly use a three‐step approach, whereas MB commonly use a two‐step quick‐attack approach) [6], these differences were treated as position‐typical task characteristics. The analysis window was therefore uniformly defined as the take‐off transition segment from right‐foot contact in the final step (T1) to bilateral toe‐off (T4) to improve comparability. Preferred position‐specific take‐off patterns were retained as part of the position‐typical spiking tasks rather than being standardized across positions.

To reduce task variability, the setter’s standing position, starting position, attacking zone, inter‐trial interval, and rest time were standardized during testing. In each trial, participants used the moment of ball release by the setter as a timing reference to execute a position‐typical approach and take‐off. A trial was deemed invalid and repeated immediately if the set deviated from the predefined area or caused an obvious mismatch in spiking timing, with substantial disruption of the contact point and movement rhythm [9]. In addition, the spike direction and landing location were constrained by a designated target area. Set height, rhythm, and trajectory, as well as ball speed and landing accuracy, were not instrumentally quantified; they were used only for task constraints and trial quality control [7, 9].

Three‐dimensional kinematic data were collected using eight Qualisys A12 infrared high‐speed cameras. Camera placement was adjusted according to the athletes’ typical approach path and take‐off location to ensure the visibility of key markers [4]. Before testing, spatial calibration was performed using a standard calibration tool, and the coordinate system was established: the *x*‐axis pointed toward the athlete’s right sideline, the *y*‐axis pointed toward the end line in the forward direction, and the *z*‐axis pointed vertically upward (Figure 1).

**Figure 1:**
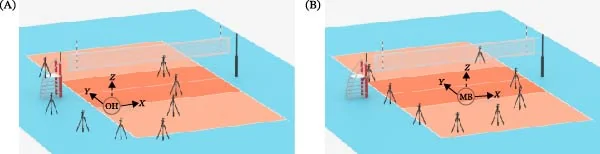
Schematic of spatial calibration. (A) Spatial calibration for the outside hitter position. (B) spatial calibration for the middle blocker position. *Note:* MB, middle blocker; OH, outside hitter.

### 2.3. Testing Procedure

After completing a 15‐min standardized warm‐up (jogging, dynamic stretching, and sport‐specific preparatory activities), participants changed into standardized testing attire and completed three familiarization trials to become accustomed to the procedure [28]. Following spatial calibration, 47 reflective markers (14 mm in diameter) were placed according to the CAST Marker Set model, and one static standing trial was recorded to establish an individualized reference model.

Surface electromyography signals were collected using a Delsys Trigno wireless system (2000 Hz). Before acquisition, skin preparation was performed following standard procedures, and electrodes were attached in accordance with the surface electromyography for noninvasive assessment of muscles (SENIAM) guidelines. Bilateral electrodes were placed over the gluteus maximus, rectus femoris, vastus medialis, vastus lateralis, semitendinosus, lateral head of the gastrocnemius, and tibialis anterior. The inter‐electrode distance was 20 mm; electrodes were aligned with the muscle fiber direction and positioned on the muscle belly and secured with medical tape.

The testing task was designed according to the athletes’ on‐court positional characteristics. For each trial, each participant first passed the ball to the setter, after which the setter delivered a standardized set. OH started from Zone 4, executed a three‐step approach and take‐off, and performed a front‐row power spike, targeting the opponent’s Zone 5 area. MB started from Zone 3, executed a two‐step quick approach and take‐off, and performed a close‐range quick attack, targeting the midline area between the opponent’s Zone 2 and Zone 4 (Figure 2).

**Figure 2:**
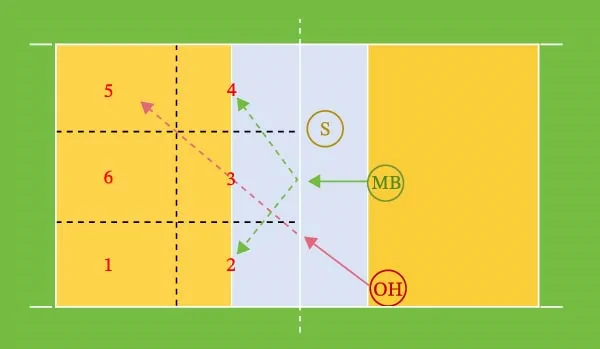
Schematic of attacking areas (S denotes the setter). *Note:* MB, middle blocker; OH, outside hitter. Solid arrows indicate the approach direction, dashed arrows indicate the attack direction.

Each participant completed six formal spike trials with a 90 s interval between trials. Three national‐level referees provided an overall score for each trial based on five criteria: approach quality, take‐off quality, timing of the spike, movement fluency, and attack effectiveness. If the set deviated from the predefined area or caused an obvious interruption of the spiking rhythm, the trial was considered invalid and repeated immediately. Ultimately, only the valid trial with the highest overall referee score was retained for each participant, and no averaging across multiple trials was performed.

The analysis window in this study was from right‐foot contact to bilateral toe‐off in the final approach step (T1–T4). Specifically, T1 was right‐foot contact (final approach step), T2 was left‐foot contact (completion of the step‐close action), T3 was peak right‐knee flexion, and T4 was bilateral toe‐off (end of take‐off) [22, 29]. T3 was used as the transition boundary marking the end of the braking phase and the beginning of the propulsion phase rather than as an independent phase. Accordingly, the braking phase was defined as T1–T3, and the propulsion phase was defined as T3–T4 (Figure 3).

**Figure 3:**
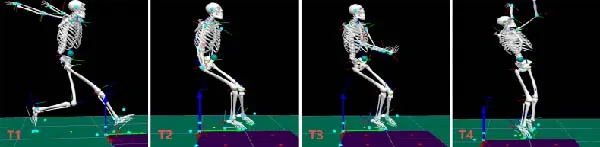
Schematic illustration of the kinematic features at the four key events (T1–T4).

### 2.4. Data Acquisition

Three‐dimensional kinematic data were collected using the Qualisys system (240 Hz), with calibration residuals controlled within 0.8 mm. After acquisition in Qualisys Track Manager (QTM), the data were imported into Visual 3D for three‐dimensional reconstruction and analysis. Bilateral hip joint angles (the included angle between the longitudinal axis of the trunk and the longitudinal axis of the thigh), knee joint angles (the included angle between the longitudinal axis of the thigh and the longitudinal axis of the shank), and ankle joint angles (the included angle between the longitudinal axis of the shank and the longitudinal axis of the dorsum of the foot) were calculated (definitions shown in Figure 4). Only sagittal‐plane flexion–extension‐related lower‐limb joint angles were analyzed in this study; frontal‐plane abduction/adduction and transverse‐plane internal/external rotation angles were not included. Joint angles were reported as geometrically‐included angles. For the knee joint, full extension (0° of conventional knee flexion) corresponds to an included angle of approximately 180°; smaller included angles indicate greater knee flexion. For the hip and ankle joints, changes in the included angles were interpreted as sagittal plane flexion–extension‐related postural changes according to the definitions shown in Figure 4. Because all participants were right‐handed hitters, side‐specific kinematic and EMG outcomes are reported as the right/dominant hitting‐side and left/non‐dominant hitting‐side values. The raw angle time series were filtered using a 10‐Hz Butterworth low‐pass filter.

**Figure 4:**
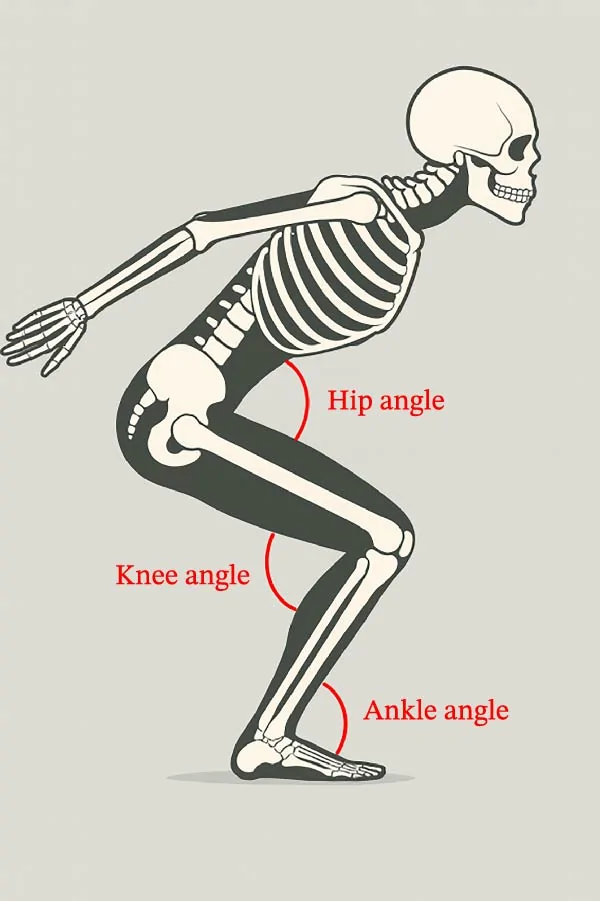
Schematic illustration of the geometric included‐angle definitions of the hip, knee, and ankle joints.

Raw EMG signals were band‐pass‐filtered (10–450 Hz) using a second‐order Butterworth filter to remove motion artifacts and high‐frequency noise, followed by full‐wave rectification; the rectified signals were then low‐pass‐filtered at 6 Hz to obtain the linear envelope. Because neither maximal voluntary contraction (MVC) nor task‐reference contraction normalization was performed, no inferences were made regarding absolute activation levels across individuals; EMG outcomes are therefore presented as the relative iEMG contribution percentage (%) within each phase [30]. EMG signals were integrated over the braking phase (T1–T3) and the propulsion phase (T3–T4) to obtain iEMG (iEMG = ∫|EMG(t)|dt, integrated over the corresponding phase duration), with T3 serving as the boundary event separating the two adjacent phase intervals. To compare distribution characteristics across muscles and sides on the same scale, “bilateral total iEMG” was used as a common denominator for each phase, defined as the sum of iEMG values of the seven muscles from both the left and right legs during that phase. The bilateral combined contribution is calculated as follows:

muscle contribution%=left iEMG of the same muscle+ right iEMG of the same muscle/bilateral total iEMG×100.

Within each phase, the summed contributions of the seven muscles were equal to 100%. The unilateral contribution is calculated as follows:

left contribution%= left iEMG of that muscle/bilateral total iEMG×100100.; right contribution%= right iEMG of that muscle/bilateral total iEMG×

The overall contribution of each leg was defined as the sum of the unilateral contributions of the seven muscles on the same side, and the overall contributions of the left and right legs sum to 100%. In these formulas, left and right correspond to the left/non‐dominant‐hitting side and right/dominant‐hitting side, respectively.

### 2.5. Statistical Analysis

Statistical analyses were performed using SPSS 22.0. Data were presented as the mean ± standard deviation (mean ± SD). The participant was treated as the statistical unit, with one selected valid trial contributing a value for each outcome variable. Normality was assessed using the Shapiro–Wilk test, and homogeneity of variance was assessed using the Levene’s test. Between‐group comparisons were conducted using independent‐samples *t*‐tests (two‐tailed, *α* = 0.05). Effect sizes (Hedges’ *g*, small‐sample corrected) and their 95% confidence intervals were reported concurrently (Tables S2–S5). Given the significant height difference between OH and MB (Table 1), to evaluate the potential impact of anthropometric differences (particularly height) on the main conclusions, an ANCOVA sensitivity analysis was further performed for key joint‐angle indicators that were directly related to the core conclusions and showed stable between‐group differences in the discrete comparisons, with height entered as a covariate (Table S1). Considering the small sample size, to avoid model over‐parameterization and reduce interpretive risk due to multiple comparisons, the sensitivity analysis was conducted on prespecified key indicators rather than applying ANCOVA to every indicator individually.

Due to constraints in recruiting elite participants, no a priori power analysis was conducted. To improve the transparency of statistical inference, a sensitivity power analysis was additionally provided (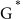
Power 3.1; independent‐samples *t* test, two‐tailed *α* = 0.05, *n* = 6 per group). The results indicate that, with the current sample size, the study is more sensitive to differences of moderate to large effects; therefore, nonsignificant outcomes should not be interpreted as “no difference” but should be interpreted in conjunction with the effect size and its 95% confidence interval (Tables S2–S5).

## 3. Results

### 3.1. Analysis of Kinematic Characteristics

This study presents both time‐normalized joint‐angle waveforms and discrete values at key events. Joint‐angle time series from T1 to T4 were normalized to 0%–100% for each participant to plot group‐mean trajectories (Figures 5–7). In these figures, the *x*‐axis represents the normalized time across the take‐off transition segment from T1 to T4, with T1 corresponding to 0% and T4 corresponding to 100%. The curves represent the normalized group‐mean waveforms across the T1–T4 window, whereas the error bars correspond to the discrete joint‐angle values at the four key events (T1–T4) and indicate ±1 sSD. Therefore, Figures 5–7 should be interpreted as time‐normalized waveform plots with key‐event discrete values rather than as peak‐value plots. Statistical inference was primarily based on between‐group comparisons of discrete joint angle values at the key events. The joint angle variables used for statistical analysis were event values at T1–T4, rather than phase‐peak or phase‐mean values.

**Figure 5:**
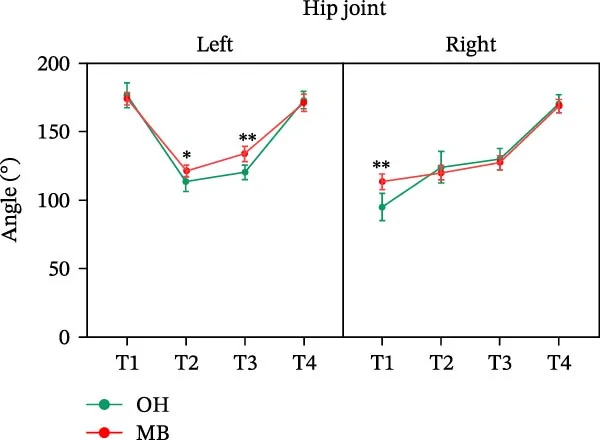
Between‐group comparisons of hip joint angles at the four key events (T1–T4). *Note:* MB, middle blocker; OH, outside hitter;  ^∗^ *p* < 0.05,  ^∗∗^ *p* < 0.01. The *x*‐axis represents normalized time across the take‐off transition segment from T1 to T4, with T1 corresponding to 0% and T4 corresponding to 100%. Curves represent the normalized group‐mean hip‐joint trajectories across the T1–T4 window, and the error bars at T1–T4 indicate ±1 standard deviation (SD) for the discrete joint‐angle values at the corresponding key events. The figure should be interpreted as a time‐normalized waveform plot with key‐event discrete values, rather than as a peak‐value plot. All participants were right‐handed hitters; therefore, the right side represents the dominant hitting side and the left side represents the non‐dominant/non‐hitting side. The terms “dominant” and “non‐dominant” refer to the hitting‐arm side rather than independently assessed lower‐limb dominance. Joint angles represent sagittal‐plane flexion–extension‐related included angles as defined in Figure 4.

**Figure 6:**
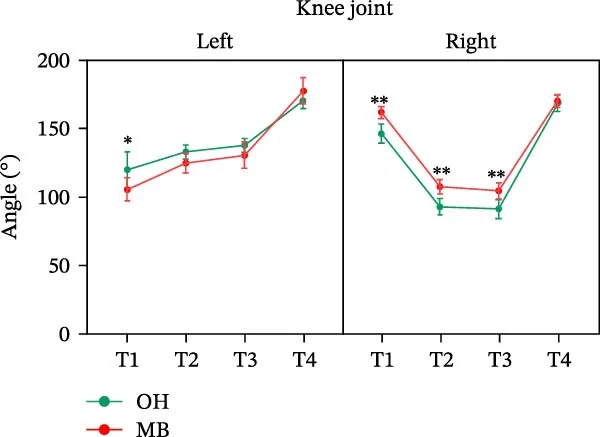
Between‐group comparisons of knee joint angles at the four key events (T1–T4). *Note:* MB, middle blocker; OH, outside hitter;  ^∗^ *p* < 0.05,  ^∗∗^ *p* < 0.01. The *x*‐axis represents normalized time across the take‐off transition segment from T1 to T4, with T1 corresponding to 0% and T4 corresponding to 100%. Curves represent the normalized group‐mean knee‐joint trajectories across the T1–T4 window, and the error bars at T1–T4 indicate ±1 standard deviation (SD) for the discrete joint‐angle values at the corresponding key events. The figure should be interpreted as a time‐normalized waveform plot with key‐event discrete values, rather than as a peak‐value plot. All participants were right‐handed hitters; therefore, the right side represents the dominant hitting side and the left side represents the non‐dominant/non‐hitting side. Joint angles represent sagittal‐plane flexion–extension‐related included angles as defined in Figure 4. For the knee joint, full extension (0° of conventional knee flexion) corresponds to an included angle of approximately 180°; smaller included angles indicate greater knee flexion.

**Figure 7:**
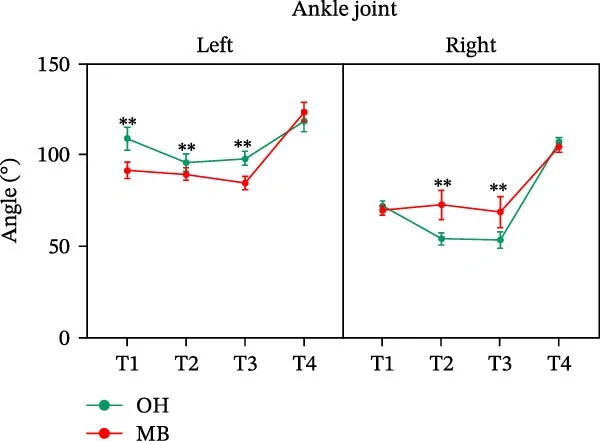
Between‐group comparisons of ankle joint angles at the four key events (T1–T4). *Note:* MB, middle blocker; OH, outside hitter;  ^∗^ *p* < 0.05,  ^∗∗^ *p* < 0.01. The *x*‐axis represents normalized time across the take‐off transition segment from T1 to T4, with T1 corresponding to 0% and T4 corresponding to 100%. Curves represent the normalized group‐mean ankle‐joint trajectories across the T1–T4 window, and the error bars at T1–T4 indicate ±1 standard deviation (SD) for the discrete joint‐angle values at the corresponding key events. The figure should be interpreted as a time‐normalized waveform plot with key‐event discrete values, rather than as a peak‐value plot. All participants were right‐handed hitters; therefore, the right side represents the dominant hitting side and the left side represents the non‐dominant/non‐hitting side. Joint angles represent sagittal‐plane flexion–extension‐related ankle included angles as defined in Figure 4.

#### 3.1.1. Hip Joint

MB exhibited significantly larger left/non‐dominant hitting‐side sagittal‐plane hip joint angles than OH at both T2 and T3, indicating a relatively more extended/open hip posture in MB and a more flexed hip posture in OH (T2: 121.06° ± 2.99 vs. 113.38° ± 7.22, *p* = 0.037; T3: 133.35° ± 5.58 vs. 119.90° ± 3.76, *p* = 0.001). Significant between‐group differences were also observed for the right/dominant hitting‐side sagittal‐plane hip joint angle in the early take‐off phase, with MB showing a significantly larger angle than OH at T1, indicating a relatively more extended/open hip posture in MB and a more flexed hip posture in OH (113.65° ± 0.78 vs. 96.00° ± 8.07, *p* = 0.001) (Figure 5).

#### 3.1.2. Knee Joint

At T1, the left/non‐dominant hitting‐side sagittal‐plane knee joint angle was significantly larger in OH than in MB, indicating a more extended knee posture in OH and greater knee flexion in MB (117.63° ± 9.25 vs. 105.64° ± 8.34, *p* = 0.040). For the right/dominant hitting‐side sagittal‐plane knee joint angle, MB showed significantly larger angles than OH at T1–T3, indicating a more extended knee posture in MB and greater right/dominant hitting‐side knee flexion in OH during this period (T1: 161.90° ± 1.55 vs. 146.43° ± 6.82, *p* = 0.001; T2: 107.54° ± 5.35 vs. 92.96° ± 5.90, *p* = 0.001; T3: 104.44° ± 5.92 vs. 91.14° ± 6.74, *p* = 0.005) (Figure 6).

#### 3.1.3. Ankle Joint

For the left/non‐dominant hitting‐side ankle, OH exhibited significantly larger sagittal‐plane ankle included angles than MB throughout T1–T3 (T1: 108.28° ± 6.02 vs. 91.03° ± 2.83, *p* = 0.001; T2: 95.36° ± 2.41 vs. 89.00° ± 3.27, *p* = 0.003; T3: 97.55° ± 1.29 vs. 84.16° ± 2.09, *p* = 0.001). For the right/dominant hitting‐side ankle, MB showed significantly larger sagittal‐plane ankle included angles than OH at T2 and T3 (T2: 72.19° ± 7.86 vs. 53.77° ± 1.20, *p* = 0.002; T3: 68.18° ± 8.46 vs. 52.99° ± 1.56, *p* = 0.002) (Figure 7).

Given the anthropometric differences (particularly height) between groups (Table 1), an ANCOVA sensitivity analysis was conducted for discrete indicators directly related to the core conclusions, with height entered as a covariate (Table S1). After controlling for height, between‐group differences remained significant for the right/dominant hitting‐side sagittal‐plane ankle included angle at T3 (*p* = 0.025) and the right/dominant hitting‐side sagittal‐plane hip joint angle at T1 (*p* = 0.014), whereas the group effect for the right/dominant hitting‐side sagittal‐plane knee joint angle at T3 was attenuated and did not reach significance (*p* = 0.064). These results suggest that the between‐group difference in the right/dominant‐hitting‐side sagittal‐plane knee joint angle at T3 tended to diminish after controlling for height.

### 3.2. Relative iEMG Contribution Percentages of Lower‐Limb Muscles in OHs and MBs

#### 3.2.1. Comparison of Bilaterally Combined Relative iEMG Contributions

Figure 8 presents the bilaterally combined relative iEMG contribution of each muscle, calculated by summing the left and right sides of the same muscle and dividing this value by the bilateral total iEMG within each phase. Therefore, Figure 8 is intended to describe the overall muscle‐level contribution pattern rather than side‐specific asymmetry; side‐specific unilateral contributions are presented separately in Figure 9. During the braking phase (Figure 8A), the primary muscles activated in OH were vastus lateralis (26.85%), vastus medialis (18.27%), and tibialis anterior (16.37%). In MB, the primary muscles activated were vastus lateralis (29.24%), vastus medialis (25.79%), and rectus femoris (10.99%). Compared with MB, OH showed higher contribution percentages for the rectus femoris (14.41% vs. 10.99%), lateral head of gastrocnemius (6.28% vs. 4.46%), and tibialis anterior (16.37% vs. 9.72%) but a lower contribution percentage for the vastus medialis (18.27% vs. 25.79%).

**Figure 8:**
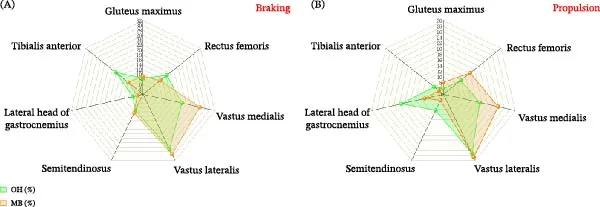
Bilaterally combined relative iEMG contribution percentages (%) of seven muscles during (A) the braking phase (T1–T3) and (B) the propulsion phase (T3–T4). *Note:* Bilaterally combined contributions were calculated by summing the left and right sides of the same muscle, with the denominator defined as the bilateral total iEMG of the seven muscles during the corresponding phase. This figure describes the overall muscle‐level contribution pattern rather than side‐specific asymmetry; side‐specific unilateral contributions are presented separately in Figure 6. MB, middle blocker; OH, outside hitter. All participants were right‐handed hitters; therefore, the right side represents the dominant hitting side and the left side represents the non‐dominant/non‐hitting side.

**Figure 9:**
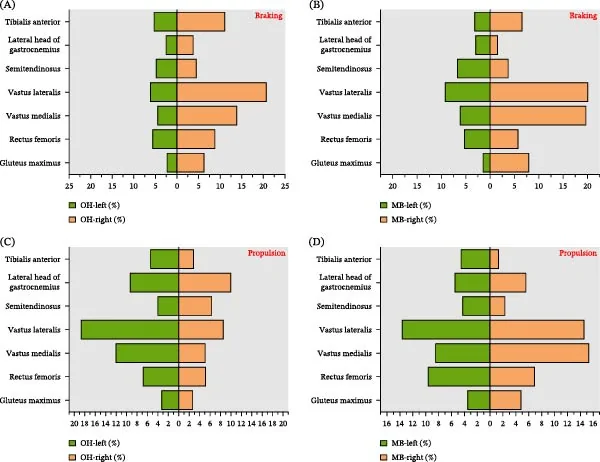
Unilateral relative iEMG contribution percentages (%) of seven muscles during the braking phase (T1–T3) and the propulsion phase (T3–T4). *Note:* Panels A–D correspond to the braking phase in outside hitters, braking phase in middle blockers, propulsion phase in outside hitters, and propulsion phase in middle blockers, respectively. Unilateral contributions were calculated by side, with the denominator defined as the bilateral total iEMG of the seven muscles during the corresponding phase. MB, middle blocker; OH, outside hitter. All participants were right‐handed hitters; therefore, the right side represents the dominant hitting side and the left side represents the non‐dominant/non‐hitting side.

During the propulsion phase (Figure 8B), the muscle with the highest contribution percentage in both groups was the vastus lateralis (OHs: 27.26% and MB: 28.67%). OHs showed higher contribution percentages for the lateral head of gastrocnemius (19.23% vs. 10.76%) and semitendinosus (10.32% vs. 6.40%), whereas MB showed higher contribution percentages for the vastus medialis (23.82% vs. 17.13%) and rectus femoris (16.03% vs. 11.88%).

#### 3.2.2. Comparison of Unilateral Relative Contributions of Integrated EMG

During the braking phase (Figure 9A,B), OH showed a dominant contribution from the right/dominant‐hitting side during the braking phase (right/dominant‐hitting side: 68.90%; left/non‐dominant‐hitting side: 31.09%). Within the right/dominant‐hitting‐side leg, the muscle with the highest contribution percentage was the vastus lateralis (20.71%), followed by the vastus medialis (13.85%) and rectus femoris (8.79%). MB showed a relatively more balanced bilateral distribution (right/dominant‐hitting side: 65.20%; left/non‐dominant‐hitting side: 34.79%), with right/dominant‐hitting‐side activation mainly concentrated in the vastus lateralis (20.02%) and vastus medialis (19.67%).

During the propulsion phase (Figure 9C,D), OH showed dominant contributions from the left/non‐dominant‐hitting side (left/non‐dominant‐hitting side: 59.40%; right/dominant‐hitting side: 40.60%). On the left/non‐dominant‐hitting side, the vastus lateralis showed the highest contribution percentage (18.65%), followed by the vastus medialis (12.03%) and lateral head of gastrocnemius (9.29%). Meanwhile, the right/dominant‐hitting‐side lateral head of gastrocnemius also showed a relatively high contribution (9.94%). In contrast, MB exhibited nearly symmetrical bilateral contributions (left/non‐dominant‐hitting side: 49.63%; right/dominant‐hitting side: 50.37%). The distributions of the primary contributing muscles by side are shown in Figure 9.

## 4. Discussion

In a position‐typical spiking context, this study synchronously compared the time‐course changes in hip–knee–ankle joint angles and the phase‐specific relative iEMG contribution percentages, aiming to characterize differences in coordination strategies between OH and MB during the spike take‐off transition segment (T1–T4) from a “kinematic–electromyographic coupling” perspective. Previous evidence on positional differences has mostly focused on overall performance outcomes such as jump height and ground contact time or on discrete indicators of a single joint [18]. Meanwhile, EMG studies show substantial heterogeneity in movement phase definition, amplitude normalization, bilateral computation conventions, and trial processing, making it difficult to systematically present the correspondence between “changes in joint angles” and “distribution of muscle activation” along the same time axis [31]. In contrast, the present study uses the critical transition window from final‐step foot contact to bilateral toe‐off as a unified temporal framework and reports joint‐angle time series and relative muscle contribution outcomes in parallel [8], providing more direct comparative evidence for understanding phase‐specific control characteristics during the braking‐to‐propulsion transition across positions.

At the kinematic level, MB showed a larger right/dominant‐hitting‐side sagittal‐plane hip joint angle in the early take‐off phase (T1), and the left/non‐dominant‐hitting‐side hip joint angle difference further increased during T2–T3, suggesting that MB maintained a relatively more “open” or extended hip posture during the transition segment, whereas OH adopted a more flexed hip posture. Such hip openness may facilitate stable control of the pelvis–trunk posture and bilateral limb coordination, thereby supporting the demands of quick attacks and block–attack transitions for rapid vertical propulsion, rhythm consistency, and postural repeatability [32]. Meanwhile, differences at the ankle joint were more pronounced at key events: during T2–T3, MB exhibited a larger right/dominant‐hitting‐side sagittal‐plane ankle included angle, whereas OH showed more evident side‐to‐side ankle differences during the braking phase [27], indicating differentiated preparatory postures and control strategies at the distal joints between positions. Considering the established view that the final step is the critical segment for horizontal‐to‐vertical momentum conversion and thus for take‐off quality [10], it can be inferred that OH in power attack situations may need to accomplish a more substantial braking‐to‐propulsion transition within a short time and preserve greater control margin for in‐air adjustments before ball contact [22, 33]; in contrast, MB may be more likely to complete take‐off with a more stable posture and more consistent rhythm to accommodate the repetitive demands of high‐frequency quick attacks and blocking [34].

It should be emphasized that a significant height difference existed between the two groups, consistent with positional roles and long‐term talent selection characteristics [16]. ANCOVA sensitivity analyses showed that, after controlling for height, between‐group differences remained stable for the right/dominant hitting‐side sagittal‐plane hip joint angle at T1 and the right/dominant hitting‐side sagittal‐plane ankle included angle at T3, whereas the group effect for the right/dominant hitting‐side sagittal‐plane knee joint angle at T3 was attenuated (*p* = 0.064). These findings suggest that the hip and ankle differences are more likely to represent relatively robust components of position‐typical control characteristics, whereas the discrete knee‐joint difference may be influenced, to some extent, by anthropometric factors. Therefore, knee‐joint mechanisms are presented here as “context‐dependent features”: in the unadjusted analysis, MB exhibited significantly larger right/dominant‐hitting‐side sagittal‐plane knee joint angles at T1–T3, indicating a more extended knee posture, whereas OH showed deeper right/dominant‐hitting‐side knee flexion during the braking phase. This difference may relate to braking depth in the final step and the eccentric control strategy of the knee extensor muscle group [29]; however, given the weakened group effect after adjustment, knee‐related interpretations should not be elevated to core evidence and are more appropriately treated as auxiliary cues in conjunction with the hip and ankle findings and EMG distribution.

Regarding EMG distribution, OH and MB exhibited markedly different “bilateral organization patterns” during the braking phase (T1–T3). OH showed a clear dominance of the right/dominant‐hitting‐side leg in overall contribution [27], with the right/dominant‐hitting‐side vastus lateralis and right/dominant‐hitting‐side vastus medialis as the leading contributors; tibialis anterior also showed a relatively high contribution during braking, suggesting that OH relied more on the knee extensor muscles and ankle dorsiflexion control for braking and postural stabilization during ground contact in the take‐off transition [12, 35, 36]. This is consistent with previous views that the quadriceps femoris plays a key eccentric control role during the braking‐to‐propulsion transition in spike‐related tasks [37]. In contrast, MB showed a more balanced bilateral distribution during braking and a more dispersed contribution structure across the quadriceps femoris (e.g., vastus lateralis, vastus medialis, and rectus femoris) [38], suggesting that MB may maintain rhythm consistency and movement repeatability through bilateral coordination and broader muscle participation, aligning with the demands of stable contacts and rapid transitions in frequent quick attack and blocking jumps [39]. It should be noted that MVC or task‐reference contraction normalization was not applied in this study; thus, the EMG findings primarily reflect within‐phase relative distribution and strategy orientation rather than between‐individual comparisons of absolute activation magnitude [30].

During the propulsion phase (T3–T4), OH exhibited a dominant propulsion distribution on the left/non‐dominant‐hitting‐side leg, whereas MB maintained an approximately symmetrical bilateral output. In both groups, the primary contributing muscles were concentrated in the vastus medialis, vastus lateralis, and the lateral head of gastrocnemius; however, OH showed a more concentrated distribution, with contributions arising from fewer muscles and predominantly from the left/non‐dominant‐hitting side, and the lateral head of gastrocnemius contributed more during OH propulsion. Considering the ankle side‐to‐side differences and the greater contribution of the lateral head of gastrocnemius, we propose the following coupling hypothesis: during the braking‐to‐propulsion transition, OH may adopt a side‐specific, phase‐dependent strategy, with right/dominant hitting‐side braking control during the braking phase and left/non‐dominant hitting‐side propulsion during the propulsion phase [22, 26], together with greater involvement of distal plantarflexor muscles to meet the demands of end‐range take‐off control and in‐air adjustments during power spikes. In contrast, MB may adopt a bilaterally coordinated strategy with a more balanced output to support stable take‐off trajectories, a consistent rhythm, and continuous task linking required for quick attacks [1]. This hypothesis aligns with prior observations that bilateral asymmetry may still exist in two‐foot take‐off and that control strategies vary under different positional task constraints [40].

It is also important to distinguish that the positional differences described above may reflect neuromuscular adaptations formed through long‐term talent selection and position‐specific training and may also, to some extent, reflect immediate constraints imposed by the “position‐typical tasks” adopted in this study on movement strategy during testing [18, 40]. Given the cross‐sectional design and the absence of a control condition with standardized step patterns and rhythm, the present study is more appropriately interpreted as describing “coordination strategy characteristics under position‐typical contexts” rather than making causal attributions to long‐term adaptation or immediate tactical choices. Future work may further separate the relative contributions of these factors through matched task comparisons or longitudinal training interventions.

From a practical perspective, the findings suggest that training priorities can be optimized according to position‐typical tasks: OH training may place greater emphasis on unilateral eccentric control, the ability to switch driving dominance between sides, and ankle–knee temporal coordination (particularly the organization during the braking‐to‐propulsion transition) [25]; MB training may place greater emphasis on bilateral synchronous force production and rhythm stability to support repeated jumping performance in high‐frequency quick attack and blocking scenarios [2, 13]. The above interpretations of coordination strategies are mainly derived from kinematic–electromyographic coupling characteristics and should be further examined in future studies incorporating ground reaction forces and inverse dynamics. Because injury outcomes were not recorded in this study, statements regarding overuse risk are limited to potential inferences; nonetheless, for athletes with pronounced side‐specific differentiation, training monitoring may still attend to changes in bilateral distribution and movement consistency, together with individualized adjustments based on sport‐specific load management [41–43].

Several limitations should be acknowledged. First, due to constraints in recruiting elite athletes, the sample size was small; the present results are primarily intended to characterize coordination features under position‐typical tasks, and the stability of effect estimates should be verified in larger samples. Second, ground reaction forces were not collected, precluding inverse dynamics calculations; thus, discussion of coordination mechanisms is mainly based on kinematics and relative iEMG distribution. Finally, although task consistency was controlled by using a fixed setter, repeating invalid trials, and selecting the highest‐rated valid trial, set parameters and attack outcomes were not instrumentally quantified. Because the analysis was based on one selected valid trial per participant rather than on multiple‐trial averages, trial‐to‐trial variability could not be assessed, and the findings may have been influenced by the selection of the highest‐rated trial. In addition, lower‐limb dominance was not independently assessed, and preferred position‐specific take‐off patterns were retained rather than standardized across positions; therefore, side‐specific findings should be interpreted within the context of position‐typical spiking tasks. Future studies may validate these findings in larger samples under standardized sets and multitrial averaging, combined with ground reaction forces and inverse dynamics; additionally, with synchronized ground reaction forces and inverse dynamics, data‐driven deep‐learning frameworks may be used to model and predict mechanisms of tissue fatigue and failure risk under repeated loading [44].

## 5. Conclusion

Based on synchronized analyses of three‐dimensional kinematics and surface electromyography, this study revealed differences in lower‐limb coordination characteristics between OH and MB during the spike‐take‐off transition segment (T1–T4). Kinematically, MB showed a larger right/dominant hitting‐side sagittal‐plane hip joint angle in the early take‐off phase, indicating a relatively more open/extended hip posture, and a larger right/dominant hitting‐side sagittal‐plane ankle included angle toward the end of the braking phase, whereas OH exhibited more pronounced side‐to‐side differences at the knee and ankle joints. After controlling for anthropometric differences (particularly height), between‐group differences at the hip and ankle joints remained generally stable, whereas the between‐group difference in the right/dominant‐hitting‐side sagittal‐plane knee joint angle at T3 was attenuated, suggesting that position‐related knee differences may be influenced, to some extent, by anthropometric factors. In terms of EMG contribution percentages, OH relied more on the right/dominant‐hitting‐side knee extensor muscle group for braking and postural control during the braking phase and showed dominant propulsion on the left/non‐dominant‐hitting side during the propulsion phase; MB exhibited a more balanced bilateral distribution during both phases. These findings provide evidence to support position‐specific strength and coordination training. However, because ground reaction forces were not collected, no direct inferences are made regarding kinetic variables such as joint moments or power, and the underlying mechanisms require further verification in future studies.

## Funding

No funding was received for this manuscript.

## Conflicts of Interest

The authors declare no conflicts of interest.

## Supporting Information

Additional supporting information can be found online in the Supporting Information section.

## Supporting information


**Supporting Information** Table S1: (angles only). Sensitivity ANCOVA controlling for height. Tables S2–S5: Effect sizes (OH vs. MB; *n* = 6 per group). Table S3: EMG bilateral relative iEMG contribution (%): OH vs. MB (Figure 8). Table S4: Leg‐level contribution (% of bilateral total iEMG): OH vs MB (Figure 9, scheme B). Table S5: Within‐group left–right unilateral contributions (% of bilateral total iEMG): Hedges’ g_z (Figure 9, scheme B).

## Data Availability

The data that support the findings of this study are available upon request from the corresponding author. The data are not publicly available due to privacy or ethical restrictions.
