# Supplementary material for: Differences in Lower‐Limb Kinematic–Electromyographic Coupling Patterns During Volleyball Spike Take‐Off Between Outside Hitters and Middle Blockers
Source: Appl Bionics Biomech. 2026 Oct 1;2026:2870235. doi: 10.1155/abb/2870235 (PMC13628560; doi:10.1155/abb/2870235)
Supplement: Supplementary file 1 [file ABB-2026-2870235-s001.docx]

**Supplementary Table S1 (angles only). Sensitivity ANCOVA controlling for height**

Notes: Group differences in three key joint-angle outcomes were tested. Primary comparisons used Welch’s t-test. Given a significant height difference between groups, an ANCOVA sensitivity analysis with height as a covariate was additionally performed. The homogeneity-of-slopes assumption was met for all outcomes (Group×Height p>0.05).

| Outcome | Unit | OH mean ± SD | MB mean ± SD | Welch t-test p | Height-adjusted ANCOVA F(1,9) | Height-adjusted ANCOVA p | partial η² | Adj mean ± SE (OH) | Adj mean ± SE (MB) |
| --- | --- | --- | --- | --- | --- | --- | --- | --- | --- |
| Right knee angle at T3 | ° | 91.14 ± 6.74 | 104.44 ± 5.92 | 0.005 | 4.461 | 0.064 | 0.331 | 91.67 ± 3.48 | 103.91 ± 3.48 |
| Right ankle angle at T3 | ° | 53.00 ± 1.56 | 68.18 ± 8.46 | 0.007 | 7.228 | 0.025 | 0.445 | 53.09 ± 3.35 | 68.08 ± 3.35 |
| Right hip angle at T1 | ° | 96.00 ± 8.07 | 113.65 ± 0.78 | 0.003 | 9.143 | 0.014 | 0.504 | 96.99 ± 3.11 | 112.67 ± 3.11 |

**Supplementary Tables S2-S5: Effect Sizes (OH vs MB; n=6 per group)**

Effect sizes are reported as Hedges’ g (between-group, independent samples) with 95% confidence intervals (CI). For within-group left–right comparisons (Fig. 9, scheme B), Hedges’ g_z (paired) with 95% CI is reported. A CI that crosses 0 indicates the effect size estimate is compatible with no difference.

**Table S2. Kinematic joint angles (°): OH vs MB (Hedges’ g with 95% CI).**

| Joint | Side | Event | OH (mean±SD) | MB (mean±SD) | Mean diff (OH−MB) | Hedges’ g | 95% CI | CI crosses 0? |
| --- | --- | --- | --- | --- | --- | --- | --- | --- |
| Hip angle (°) | Left | T1 | 176.18 ± 8.88 | 173.71 ± 4.41 | 2.47 | 0.33 | [-0.81, 1.47] | Yes |
| Hip angle (°) | Left | T2 | 113.38 ± 7.22 | 121.06 ± 2.99 | -7.68 | -1.28 | [-2.55, -0.02] | No |
| Hip angle (°) | Left | T3 | 119.90 ± 3.76 | 133.35 ± 5.58 | -13.45 | -2.61 | [-4.22, -1.00] | No |
| Hip angle (°) | Left | T4 | 172.58 ± 5.33 | 170.83 ± 6.28 | 1.75 | 0.28 | [-0.86, 1.42] | Yes |
| Hip angle (°) | Right | T1 | 96.00 ± 8.07 | 113.65 ± 0.78 | -17.65 | -2.84 | [-4.52, -1.16] | No |
| Hip angle (°) | Right | T2 | 124.25 ± 8.48 | 119.63 ± 0.38 | 4.62 | 0.71 | [-0.46, 1.88] | Yes |
| Hip angle (°) | Right | T3 | 129.58 ± 5.45 | 126.83 ± 2.35 | 2.75 | 0.60 | [-0.56, 1.77] | Yes |
| Hip angle (°) | Right | T4 | 170.02 ± 3.76 | 168.25 ± 3.44 | 1.77 | 0.45 | [-0.70, 1.60] | Yes |
| Knee angle (°) | Left | T1 | 117.63 ± 9.25 | 105.64 ± 8.34 | 11.99 | 1.26 | [-0.00, 2.52] | Yes |
| Knee angle (°) | Left | T2 | 132.78 ± 5.15 | 125.11 ± 7.37 | 7.67 | 1.11 | [-0.12, 2.35] | Yes |
| Knee angle (°) | Left | T3 | 137.67 ± 5.11 | 130.53 ± 9.73 | 7.14 | 0.85 | [-0.34, 2.04] | Yes |
| Knee angle (°) | Left | T4 | 170.46 ± 5.79 | 177.71 ± 9.36 | -7.25 | -0.86 | [-2.05, 0.33] | Yes |
| Knee angle (°) | Right | T1 | 146.43 ± 6.82 | 161.90 ± 1.55 | -15.47 | -2.89 | [-4.59, -1.19] | No |
| Knee angle (°) | Right | T2 | 92.96 ± 5.90 | 107.54 ± 5.35 | -14.58 | -2.39 | [-3.93, -0.85] | No |
| Knee angle (°) | Right | T3 | 91.14 ± 6.74 | 104.44 ± 5.92 | -13.30 | -1.94 | [-3.35, -0.52] | No |
| Knee angle (°) | Right | T4 | 168.66 ± 6.26 | 170.08 ± 4.59 | -1.42 | -0.24 | [-1.38, 0.90] | Yes |
| Ankle angle (°) | Left | T1 | 108.28 ± 6.02 | 91.03 ± 2.83 | 17.25 | 3.39 | [1.52, 5.25] | No |
| Ankle angle (°) | Left | T2 | 95.36 ± 2.41 | 89.00 ± 3.27 | 6.36 | 2.04 | [0.60, 3.49] | No |
| Ankle angle (°) | Left | T3 | 97.55 ± 1.29 | 84.16 ± 2.09 | 13.39 | 7.12 | [3.80, 10.44] | No |
| Ankle angle (°) | Left | T4 | 117.82 ± 5.67 | 122.92 ± 5.15 | -5.10 | -0.87 | [-2.06, 0.32] | Yes |
| Ankle angle (°) | Right | T1 | 71.40 ± 2.66 | 69.23 ± 2.57 | 2.17 | 0.77 | [-0.41, 1.95] | Yes |
| Ankle angle (°) | Right | T2 | 53.77 ± 1.20 | 72.19 ± 7.86 | -18.42 | -3.02 | [-4.77, -1.28] | No |
| Ankle angle (°) | Right | T3 | 52.99 ± 1.56 | 68.18 ± 8.46 | -15.19 | -2.31 | [-3.82, -0.79] | No |
| Ankle angle (°) | Right | T4 | 106.48 ± 2.23 | 103.94 ± 3.02 | 2.54 | 0.88 | [-0.31, 2.08] | Yes |

**Table S3. EMG bilateral relative iEMG contribution (%): OH vs MB (Fig. 8).**

*Bilateral contribution for muscle m: (iEMG_L + iEMG_R)/Σ(iEMG over 7 muscles and both legs) × 100%.*

| Phase | Variable | OH (mean±SD) | MB (mean±SD) | Mean diff (OH−MB) | Hedges’ g | 95% CI | CI crosses 0? |
| --- | --- | --- | --- | --- | --- | --- | --- |
| Braking (T1–T3) | GM | 8.50 ± 3.13 | 9.36 ± 1.27 | -0.85 | -0.33 | [-1.47, 0.81] | Yes |
| Braking (T1–T3) | RF | 14.42 ± 3.38 | 10.99 ± 1.20 | 3.42 | 1.25 | [-0.01, 2.50] | Yes |
| Braking (T1–T3) | VM | 18.27 ± 1.82 | 25.79 ± 1.92 | -7.53 | -3.71 | [-5.70, -1.73] | No |
| Braking (T1–T3) | VL | 26.85 ± 2.83 | 29.23 ± 1.41 | -2.38 | -0.99 | [-2.20, 0.23] | Yes |
| Braking (T1–T3) | ST | 9.30 ± 1.99 | 10.44 ± 1.30 | -1.14 | -0.62 | [-1.79, 0.54] | Yes |
| Braking (T1–T3) | LG | 6.28 ± 0.90 | 4.46 ± 0.51 | 1.82 | 2.30 | [0.78, 3.81] | No |
| Braking (T1–T3) | TA | 16.38 ± 1.88 | 9.73 ± 1.07 | 6.65 | 4.01 | [1.92, 6.10] | No |
| Propulsion (T3–T4) | GM | 5.93 ± 1.25 | 8.23 ± 1.22 | -2.30 | -1.72 | [-3.08, -0.36] | No |
| Propulsion (T3–T4) | RF | 11.88 ± 1.97 | 16.03 ± 2.16 | -4.15 | -1.85 | [-3.25, -0.46] | No |
| Propulsion (T3–T4) | VM | 17.12 ± 1.49 | 23.82 ± 1.68 | -6.70 | -3.90 | [-5.95, -1.85] | No |
| Propulsion (T3–T4) | VL | 27.26 ± 2.42 | 28.67 ± 3.93 | -1.41 | -0.40 | [-1.54, 0.75] | Yes |
| Propulsion (T3–T4) | ST | 10.32 ± 3.70 | 6.40 ± 1.90 | 3.92 | 1.23 | [-0.02, 2.49] | Yes |
| Propulsion (T3–T4) | LG | 19.23 ± 1.26 | 10.76 ± 1.91 | 8.47 | 4.83 | [2.43, 7.23] | No |
| Propulsion (T3–T4) | TA | 8.25 ± 3.23 | 6.09 ± 2.28 | 2.16 | 0.71 | [-0.46, 1.89] | Yes |

**Table S4. Leg-level contribution (% of bilateral total iEMG): OH vs MB (Fig. 9, scheme B).**

*Leg contribution is the sum of unilateral contributions of the 7 muscles on the same leg; Left + Right = 100%.*

| Phase | Side | OH (mean±SD) | MB (mean±SD) | Mean diff (OH−MB) | Hedges’ g | 95% CI | CI crosses 0? |
| --- | --- | --- | --- | --- | --- | --- | --- |
| Braking (T1–T3) | Left | 31.09 ± 2.01 | 34.80 ± 1.33 | -3.71 | -2.01 | [-3.44, -0.57] | No |
| Braking (T1–T3) | Right | 68.91 ± 2.01 | 65.20 ± 1.33 | 3.71 | 2.01 | [0.57, 3.44] | No |
| Propulsion (T3–T4) | Left | 59.40 ± 4.15 | 49.62 ± 5.73 | 9.78 | 1.80 | [0.42, 3.18] | No |
| Propulsion (T3–T4) | Right | 40.60 ± 4.15 | 50.38 ± 5.73 | -9.78 | -1.80 | [-3.18, -0.42] | No |

**Table S5. Within-group left–right unilateral contributions (% of bilateral total iEMG): Hedges’ g_z (Fig. 9, scheme B).**

*Unilateral contribution: iEMG_{m,side}/Σ(iEMG over 7 muscles and both legs) × 100%.*

| Group | Phase | Variable | Left (mean±SD) | Right (mean±SD) | Mean diff (L−R) | Hedges’ g_z | 95% CI | CI crosses 0? |
| --- | --- | --- | --- | --- | --- | --- | --- | --- |
| OH | Braking (T1–T3) | Leg total | 31.09 ± 2.01 | 68.91 ± 2.01 | -37.82 | -7.92 | [-12.89, -2.95] | No |
| OH | Braking (T1–T3) | GM | 2.29 ± 1.10 | 6.22 ± 2.55 | -3.93 | -1.39 | [-2.57, -0.21] | No |
| OH | Braking (T1–T3) | RF | 5.62 ± 0.55 | 8.79 ± 3.23 | -3.17 | -0.84 | [-1.80, 0.11] | Yes |
| OH | Braking (T1–T3) | VM | 4.42 ± 1.25 | 13.85 ± 2.48 | -9.43 | -2.29 | [-3.91, -0.66] | No |
| OH | Braking (T1–T3) | VL | 6.14 ± 0.75 | 20.71 ± 2.69 | -14.58 | -4.45 | [-7.32, -1.58] | No |
| OH | Braking (T1–T3) | ST | 4.80 ± 1.36 | 4.50 ± 1.05 | 0.30 | 0.18 | [-0.63, 0.98] | Yes |
| OH | Braking (T1–T3) | LG | 2.52 ± 0.37 | 3.76 ± 0.74 | -1.24 | -1.39 | [-2.57, -0.22] | No |
| OH | Braking (T1–T3) | TA | 5.30 ± 1.38 | 11.07 ± 1.97 | -5.77 | -1.71 | [-3.04, -0.38] | No |
| OH | Propulsion (T3–T4) | Leg total | 59.40 ± 4.15 | 40.60 ± 4.15 | 18.80 | 1.91 | [0.48, 3.33] | No |
| OH | Propulsion (T3–T4) | GM | 3.28 ± 0.57 | 2.66 ± 1.00 | 0.62 | 0.50 | [-0.36, 1.36] | Yes |
| OH | Propulsion (T3–T4) | RF | 6.79 ± 0.77 | 5.09 ± 2.02 | 1.70 | 0.61 | [-0.28, 1.49] | Yes |
| OH | Propulsion (T3–T4) | VM | 12.03 ± 1.10 | 5.10 ± 0.97 | 6.93 | 4.01 | [1.40, 6.63] | No |
| OH | Propulsion (T3–T4) | VL | 18.65 ± 2.06 | 8.61 ± 1.25 | 10.04 | 3.53 | [1.20, 5.86] | No |
| OH | Propulsion (T3–T4) | ST | 3.98 ± 0.85 | 6.34 ± 3.43 | -2.36 | -0.59 | [-1.47, 0.29] | Yes |
| OH | Propulsion (T3–T4) | LG | 9.29 ± 1.04 | 9.94 ± 0.86 | -0.65 | -0.38 | [-1.22, 0.45] | Yes |
| OH | Propulsion (T3–T4) | TA | 5.38 ± 2.44 | 2.86 ± 1.04 | 2.52 | 1.12 | [0.06, 2.18] | No |
| MB | Braking (T1–T3) | Leg total | 34.80 ± 1.33 | 65.20 ± 1.33 | -30.41 | -9.62 | [-15.64, -3.61] | No |
| MB | Braking (T1–T3) | GM | 1.39 ± 0.35 | 7.96 ± 1.06 | -6.57 | -5.95 | [-9.73, -2.18] | No |
| MB | Braking (T1–T3) | RF | 5.25 ± 0.89 | 5.74 ± 1.06 | -0.49 | -0.27 | [-1.08, 0.55] | Yes |
| MB | Braking (T1–T3) | VM | 6.12 ± 0.55 | 19.67 ± 1.53 | -13.56 | -9.00 | [-14.64, -3.37] | No |
| MB | Braking (T1–T3) | VL | 9.22 ± 1.76 | 20.02 ± 1.76 | -10.80 | -2.82 | [-4.74, -0.90] | No |
| MB | Braking (T1–T3) | ST | 6.70 ± 0.96 | 3.74 ± 0.67 | 2.96 | 2.43 | [0.72, 4.13] | No |
| MB | Braking (T1–T3) | LG | 2.95 ± 0.28 | 1.51 ± 0.56 | 1.44 | 1.69 | [0.37, 3.00] | No |
| MB | Braking (T1–T3) | TA | 3.16 ± 0.64 | 6.56 ± 1.03 | -3.40 | -2.13 | [-3.68, -0.59] | No |
| MB | Propulsion (T3–T4) | Leg total | 49.62 ± 5.73 | 50.38 ± 5.73 | -0.76 | -0.06 | [-0.86, 0.75] | Yes |
| MB | Propulsion (T3–T4) | GM | 3.54 ± 0.70 | 4.69 ± 1.58 | -1.15 | -0.46 | [-1.31, 0.39] | Yes |
| MB | Propulsion (T3–T4) | RF | 9.35 ± 0.77 | 6.68 ± 2.84 | 2.66 | 0.63 | [-0.26, 1.52] | Yes |
| MB | Propulsion (T3–T4) | VM | 8.49 ± 1.15 | 15.33 ± 2.13 | -6.84 | -1.93 | [-3.37, -0.49] | No |
| MB | Propulsion (T3–T4) | VL | 14.08 ± 4.02 | 14.59 ± 1.12 | -0.51 | -0.10 | [-0.90, 0.71] | Yes |
| MB | Propulsion (T3–T4) | ST | 4.08 ± 1.94 | 2.32 ± 0.26 | 1.75 | 0.73 | [-0.19, 1.65] | Yes |
| MB | Propulsion (T3–T4) | LG | 5.34 ± 0.77 | 5.42 ± 1.26 | -0.09 | -0.09 | [-0.89, 0.71] | Yes |
| MB | Propulsion (T3–T4) | TA | 4.75 ± 2.24 | 1.34 ± 0.11 | 3.42 | 1.30 | [0.16, 2.43] | No |
